# Passenger-surface microbiome interactions in the subway of Mexico City

**DOI:** 10.1371/journal.pone.0237272

**Published:** 2020-08-19

**Authors:** Daniela Vargas-Robles, Carolina Gonzalez-Cedillo, Apolinar M. Hernandez, Luis D. Alcaraz, Mariana Peimbert

**Affiliations:** 1 Departamento de Ciencias Naturales, Unidad Cuajimalpa, Universidad Autónoma Metropolitana, Ciudad de México, México; 2 Departamento de Biología Celular, Facultad de Ciencias, Universidad Nacional Autónoma de México, Ciudad de México, México; Wilfrid Laurier University, CANADA

## Abstract

Interaction between hands and the environment permits the interchange of microorganisms. The Mexico City subway is used daily by millions of passengers that get in contact with its surfaces. In this study, we used 16S rRNA gene sequencing to characterize the microbiomes of frequently touched surfaces and compare regular and women-only wagons. We also explored the effect of surface cleaning on microbial resettling. Finally, we studied passenger behavior and characterized microbial changes after traveling. Most passengers (99%), showed some type of surface interaction during a wagon trip, mostly with the hands (92%). We found microbiome differences associated with surfaces, probably reflecting diverse surface materials and usage frequency. The platform floor was the most bacterial diverse surface, while the stair handrail and pole were the least diverse ones. After pole cleaning, the resettling of microbial diversity was fast (5–30 minutes); however, it did not resemble the initial composition. After traveling, passengers significantly increased their hand microbial diversity and converged to a similar microbial composition among passengers. Additionally, passenger hand microbiomes resembled subway surfaces in diversity. However, microbial fingerprints were preserved within passengers after traveling.

## Introduction

Mexico City’s subway transports around 1,678 million passengers per year (4.2 million daily), making it the ninth-largest transit subway in the world [1]. This high number of visitors promotes multiple physical interactions, becoming an essential system for studying colonization and disseminating microbes.

Hands are an essential channel of the interactions of humans with their surroundings. Actinobacteria, Proteobacteria, and Firmicutes are the phyla that mainly comprise the hand microbiomes [2]. They are very diverse, surpassing oral and intestinal microbiomes [3]. They highly vary among individuals and between the right and the left hand of the same person [4]. Constant exposure to diverse environmental sources and perturbations (e.g., handwashing) is a factor of the hand’s microbiome heterogeneity [4].

Interaction between hands and the environment permits the interchange of microorganisms, explaining the high proportion of human-associated microbes in built environments [5]. There is a human microbiome signal that is strong and traceable among people and buildings [6]. Cohabitation results in closer microbial composition than kinship; people sharing the same house have more similar microbial profiles than others [5]. Additionally, different types of surfaces in one home can be more similar in microbiome composition than in different houses [7]. Similarly, there are microbe differences in built areas used exclusively by women or men; for example, *Lactobacillus iners* was found as a female-associated bacterium, while *Dermabacter hominis*, *Facklamia*, and *Corynebacterium* were more abundant in rooms used by males [4,8–10]. There is evidence that indoor and human microbiomes are closely related and influence each other.

The Mexico City subway contains different microenvironments [11]. Most train lines are devoid of sunlight, and only a few lines may run in the exterior a fraction of their route. Exterior air is ventilated into the subway from a ducted air stream, and indoor air is recirculated using exhaust fans. Particulate matter levels are higher inside stations than outdoors [12]. In the hot season, water is spread out into the air by fans. While the statin floors are cleaned daily, train wagons are deep-cleaned once a month. Other surfaces such as turnstiles, stairs, and escalator handrails are cleaned eventually with a not strict schedule.

The subway is the most used transportation system in Mexico City. Subway travelers in Mexico City are exposed to the sale and consumption of food inside the premises, street vendors, and the absence of seats at the stations. The first two train cars are exclusively for women, the disabled, and the elderly [13]. Some cities have applied exclusive cars for women as a measure to decrease sexual harassment [14,15].

There are culture-independent studies of subway surfaces of New York City [16], Boston [17], Oslo [18], Mexico City [19], and by MetaSUB [20], an international initiative. Such studies have shown that the subway microbiome is structured mostly by commensal bacteria from the skin and that microbial composition and diversity vary according to the material and type of usage. In the Hong Kong subway, there are differences between morning and afternoon microbial composition, with more antibiotic resistance genes in the afternoon [21]. In the same study, the commuters’ hand microbiomes were explored measuring bacteria acquisition in a 30-minute trip [21].

**Fig 1:**
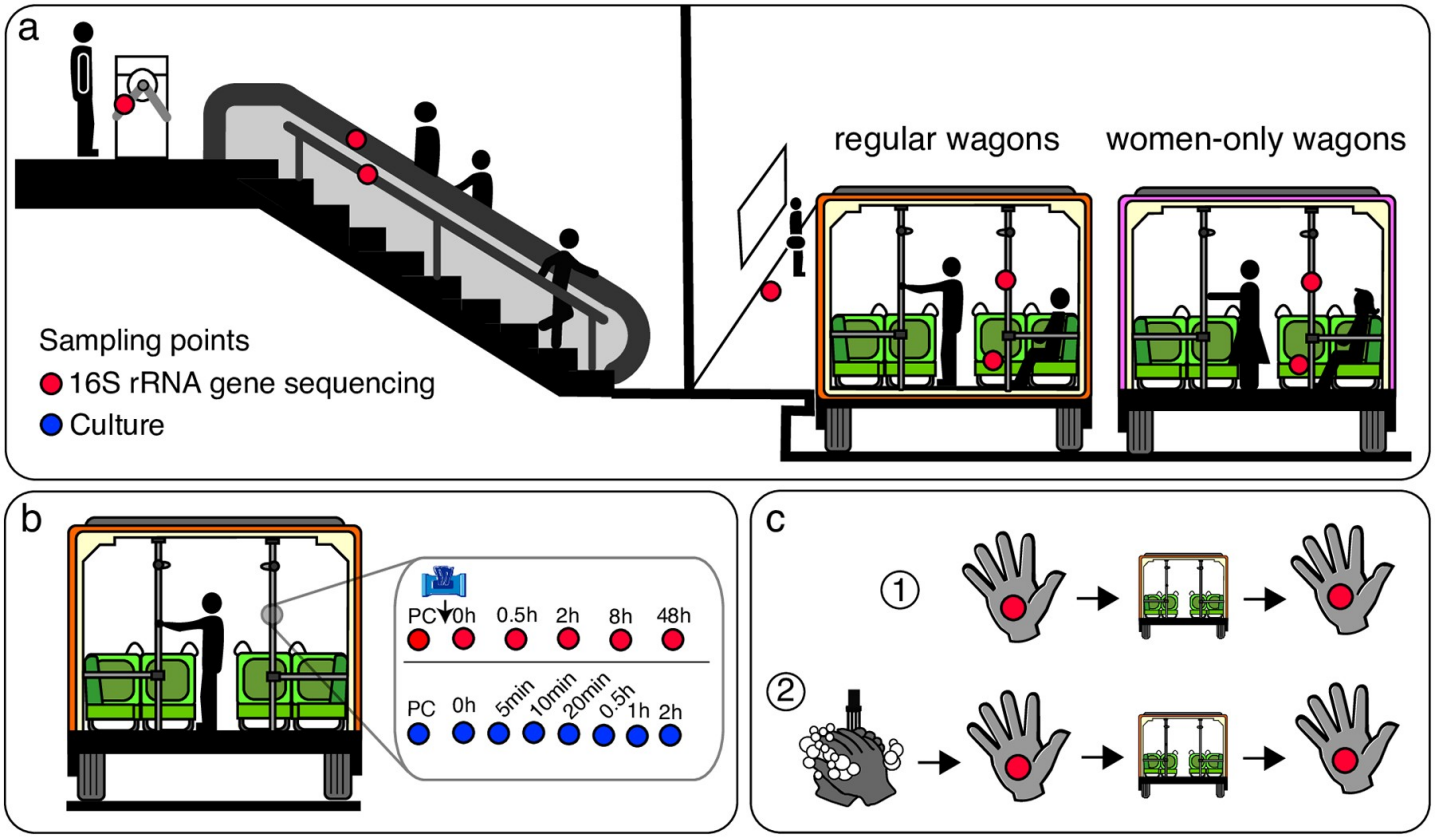
Study design. We swabbed surfaces and used the 16S rRNA gene (red dots) and microbial cultures (blue dots) to describe bacterial diversity. (a) Microbiome comparison of subway surfaces: turnstiles, escalator handrails, stair handrails, platform floors, poles, and train seats. Poles and train seats were sampled in regular and women-only wagons (N = 5 per site). (b) Microbiome succession study after a cleaning event in poles. Microbiome changes were evaluated at pre-cleaning (PC) and 0, 0.5, 2, 8, and 48 h after cleaning. (c) Hand microbial diversity before and after traveling; we evaluated the effect of traveling with and without previous handwashing.

The present study describes the interaction between the Mexico City subway microbiota and those of its passengers. We compared microbiomes from different subway surfaces comprising stations and regular and women-only wagons. We also evaluated the velocity of the bacterial succession after an event of surface cleaning. Additionally, we characterized the passengers’ microbiomes before and after traveling (Fig 1 and S1 Table).

## Methods

### Ethics statement

This study was approved by the Commission of Academic Ethics and Scientific Responsibility of the Facultad de Ciencias, Universidad Nacional Autónoma de México (P1202002001). Written informed consent was obtained from the volunteers who donated the hand swabs. All the volunteers were graduate students over 18 years old.

### Sampling

Sampling was performed in autumn 2018. Samples were taken from turnstiles, stair handrails, escalator handrails, platform floor, train poles, and seats. The latter two were sampled from two different train wagons: the regular wagon (used by men and women) and the women-only wagon (used by women, disabled people, and the elderly). The first wagons are women-only, so they change every time the train arrives at a terminal station. A total of 97 samples were collected, and 89 were successfully processed (S1 Table). Sampling was performed by swabbing each surface of around 100 cm^2^ for 20 seconds with a pre-moistened nylon-flocked swab (COPAN FLOQSwabs^™^), and samples were preserved in transport media (Tris 20 mM, EDTA 10 mM pH 7.5). Samples were kept in ice for less than 12 h until freezing at -80°C. Line and station names, time, temperature, and relative humidity were registered. Sampling permits were granted by the subway “User Support Manager” (*Gerencia de Atención al Usuario del Sistema de Transporte Colectivo*).

The impact of a subway trip on the passengers’ microbiomes was determined by swabbing the right hand of eight informed volunteers. Volunteers were sampled before and after traveling on a regular weekday. Subjects arrived at the starting point in the morning, not using the subway as a transportation means. The subway trip included traveling 11 stations across three different subway lines, including two-line transferences. It was a circular route so that they would arrive at the starting point. Each volunteer was indicated to contact particular surfaces (at least twice) and avoid touching others. Surfaces touched by each volunteer are summarized in S2 and S3 Tables. Hands were sampled after completing the trip. Additionally, volunteers were asked to wash their hands for 30 s with liquid soap (DIAL^®^ neutral) and distilled water following the same protocol. Immediately after this, hands were sampled and then resampled at the end of the trip.

### Surfaces cleaning

To describe the microbiome colonization, we cleaned five poles from the same wagon (mixed wagon) on a regular weekday morning. Areas to sample were defined with a template divided into five areas of 100 cm^2^ each. Samples were taken before and after cleaning (pre- and post-cleaning). A Lysol wipe was used to scrub the surface energetically, and then, a wet sterile gauze was used to remove the cleaning product excess. Post-cleaning samples were swabbed immediately after the cleaning of each surface. The remaining samples were taken longitudinally at five time-points (0, 0.5, 2, 8, and 48 h). We did not sample twice in the same area to avoid affecting the microbiome composition of the following time points.

Surface cleaning was also analyzed by CFU counting. As described above, we sampled 10–12 poles in seven post-cleaning time points: 0 h, 5 min, 10 min, 20 min, 0.5 h, 1 h, and 2 h. Two positive controls were also swabbed: pre-cleaning samples, and 2 h without cleaning. Bacteria were incubated in the LB-agar medium for 36 h at 30°C.

### Observational patterns during a subway trip

A total of 120 passengers (67 adults and 53 elderly) were randomly picked and observed from the start to the end of each trip. All interactions with their environment were documented during traveling. Additionally, observations were also made in the stations. The number of passengers touching the handrails of escalators and stairs was documented in 5 and 11 different stations, respectively. Differences between stairs going up or down were also explored, while the chosen escalators were only going up.

### DNA extraction

Metagenomic DNA was extracted using a MoBio PowerSoil Kit (MoBio Laboratories, Solana Beach, CA) with small modifications. Half of the beat material from each tube was poured into a new one. Volumes were adjusted to preserve proportions of solutions/samples. In total, 125 μL of the sample was used, 30 μL of C1 solution, and 50 μL of phenol: chloroform 1:1 were mixed in the beat tube. Further steps were performed according to the instructions of the MoBio PowerSoil Kit.

Region V3-V4 from the 16S rRNA gene was amplified using primers 341F (CCTACGGGNGGCWGCAG) and 805R (GACTACHVGGGTATCTAATCC). Libraries were obtained following the MiSeq^™^ Illumina^®^ protocol. Samples without PCR amplification were discarded. All samples corresponded to the ones taken just after cleaning the surfaces. The PCR was performed in triplicates, using 0.15 ul of Phusion DNA polymerase^™^, 3 μL Buffer 5x, 2.5 μL dNTP (3 mM), 1 μL forward primer (5 pmol/μL), 1 μL reverse primer (5 pmol/μL), 1–4 μL DNA, and water up to the final volume of 15 ul per reaction. The PCR reaction was initiated at 98°C, 30 s, followed by 35 cycles of 92°C for 10 s, 53°C for 30 s, 72°C for 40 s, and a final extension step at 72°C for 5 min. Blank samples were used as negative controls. The three PCR reactions per sample were combined and purified using the High Pure PCR Product Purification Kit of ROCHE^™^ (Roche Diagnostics GmbH, Mannheim, Germany). Sequencing was performed using the MiSeq^™^ Illumina^®^ (2 x 300 bp) platform at the Laboratory of Genomic Services from the National Laboratory of Genomics for Biodiversity in Irapuato, México. The DNA concentration was measured with a NanoDrop microvolume spectrophotometer.

### Bioinformatics processing

Amplified reads were pair-ended using the Context-Aware Scheme for Paired-End Read (CASPER) [22]. Sequences were clustered at 97% of identity using cd-hit-est [23], and pick_rep_set.py from QIIME (v. 1.9) [24] was used to pick representative sequences from each cluster. Chimera sequences and singletons were removed. The taxonomy assignment was done with QIIME (v. 1.9) [24] using parallel BLAST [25] and the GreenGenes database [26]. Finally, chloroplasts and mitochondria were filtered from the OTU table. These steps were processed with default parameters, and the analyzed samples were rarified at 6,242 sequences per sample.

Additionally, amplicon sequence variants (ASVs) were generated using the DADA2 algorithm [27]. Reads 341F-804R were extracted from the 16S Silva database [28] to train a Naive Bayes classifier [29].

### Data analysis

Data analysis and plot generation were performed using phyloseq [30] and ggplot2 [31] in R version 3.5.1 [32]. Beta diversity was visualized with canonical analysis of principal coordinates (CAP) and non-metric dimensional scaling (NMDS) with Bray Curtis dissimilarities. Comparison among groups was performed with the vegan package [33] from R, using the adonis function, which conducts a permutational multivariate analysis of variance (PERMANOVA) using distance matrices with 999 permutations. Group dispersion was examined with multivariate homogeneity of group dispersions with the betadisp R function. PERMANOVA pairwise comparison was performed with the pairwise.adonis function [34] in the devtools package with default parameters and adjusted p values with the false discovery rate (FDR) method. Non-parametric comparisons were performed with the Kruskal-Wallis test and pairwise comparison with Dunn’s Test of Multiple Comparisons Using Rank Sums, Dunn.test function from the R base library. The non-parametric two-group comparison was performed with the Wilcoxon test. Discriminant taxa analysis among groups was performed using the LEfSe algorithm with one against all strategy, set with an LDA inferior limit of 4 and alpha value of 0.05 [35].

## Results

### Passenger behavior and subway interaction

To understand the type and level of interaction that a passenger has with the subway environment, we traveled with passengers and registered their behavior during eight different weekdays. A total of 120 passengers were randomly picked and observed during a train trip (67 adults and 53 elders), from the entrance to the end of their trips (S4 Table). Prevalence (% [CI_95%_]) and median frequency (times/10 min) of contact with particular surfaces or objects were registered. Most of the passengers (99% [95–100%]) had contact with any wagon surface; 92% [85–96%] of the contacts were with the hands and the rest with the body or cloth. As expected, the hands were the most common means of interaction with train surfaces (4.1 times/10 min), with higher frequency in the older people than in adults (5.0 vs. 3.6 times/10 min; *p* = 0.030, Wilcoxon test). Most users touched train poles (89% [82–94%]), being the most recurrent touched surface (marginally higher in the elderly than in adults, 4.9 vs. 4.1 times/10 min; *p* = 0.051, Wilcoxon test). The passenger’s self-contact was measured, and the face/head was the most commonly touched body part (73% [64–81%], times/10 min), similar in frequency between age groups. In face/head area touching, the skin predominated (68% [58–76%], 1.3 times/10 min), followed by the hair/scalp (27% [19–36%], median of 0, mean of 0.7 times/10 min), any mucosa (17% [11–25%], median of 0, mean of 0.2 times/10 min), and the ear canal (4% [1.5–9.9%], median of 0, mean of 0.04 times/10 min), similar between age groups. Passenger’s hands were also in frequent contact with personal articles (73% [64–80%], 1.7 times/10 min), with cell phones being the most commonly and frequently used items in adults compared to the elderly (49% [37–62%] vs. 5.7% [1.5–17%], a median of 0 for both groups and mean of 0.9 vs. 0.05 times/10 min respectively; *p* = 3 x 10^−7^, Wilcoxon test). Other activities not directly related to hands, such as sitting, were also higher in the elderly (*p* = 0.001, Wilcoxon test), as well as laying the body on any other surface than seats (*p* = 0.049, Wilcoxon test). Further activities with microbiological relevance were also registered but observed to a lesser extent, such as touching other people, money interchange (buying or charity), coughing, drinking, and eating with bare hands. Although not included in S4 Table, putting on makeup, book reading, and sleeping were also eventually observed.

Additionally, we observed passengers using the escalator and stairs in the stations (N = 7,456 passengers, S5 Table). Escalator handrails were touched continuously with the hands (86.2%, N = 2,650), while stair handrails were less frequently used (20.3%, N = 4,806), being higher for stairs going down than going up (23.6% vs. 17.1% respectively, *p* < 1 x 10^−7^, Chi-squared test).

### Massive sequencing of the 16S rRNA gene

We sequenced 89 samples, with 10,538,220 reads being obtained, which resulted in 5,238,317 paired sequences of an average length of 450 bp (S6 and S7 Tables). Sequences were filtered to discard singleton, chloroplast, or mitochondrial sequences. With an average of 34,125 sequences per sample, we identified a total of 75,914 97% OTUs (1,121 genera). All samples were rarefied at the minimum sequence number per sample (6,242 sequences). Subsampling generated 29,811 OTUs (939 genera; S7 Table). A rarefied data set was used to present the results of this study. We did not identify archaeal OTUs, and only 28 OTUs (0.004% of the sequences) did not match any known organism.

### Subway surface microbiome

**Fig 2:**
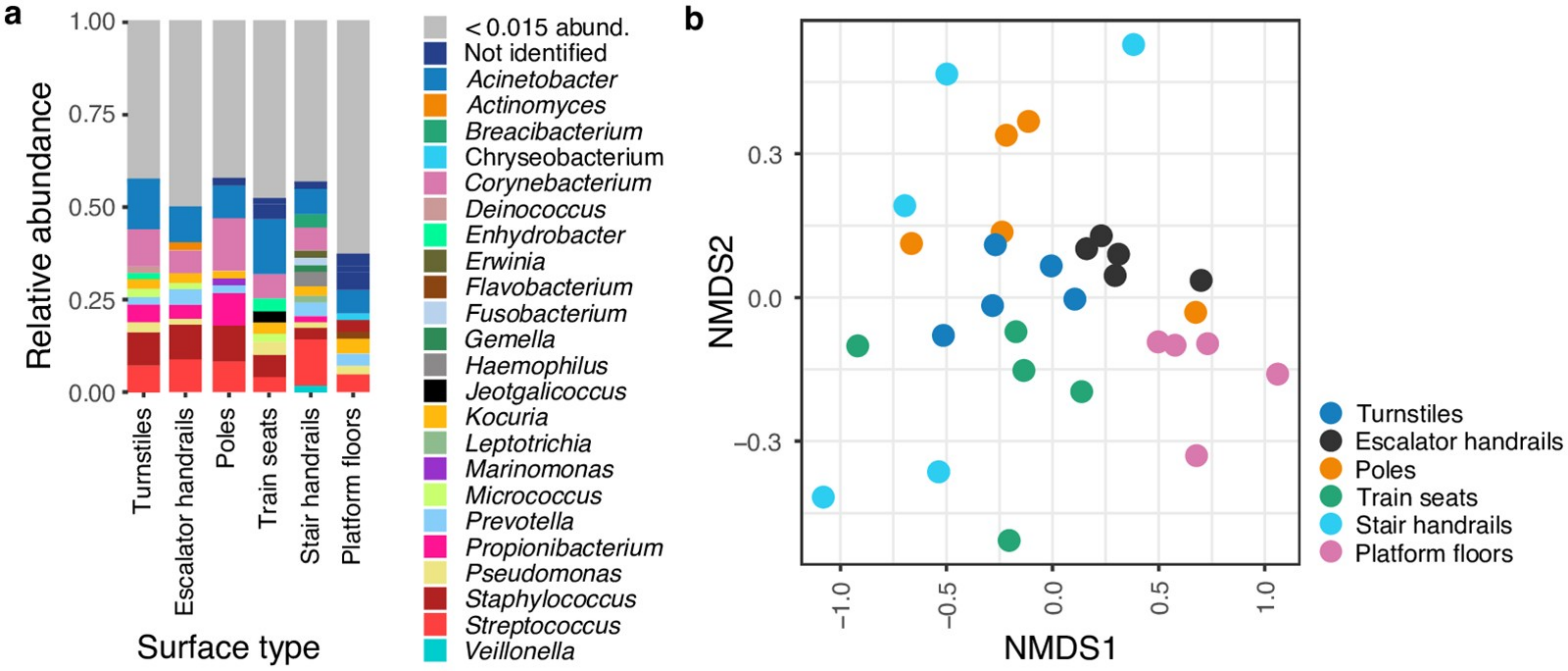
Subway surface microbiome diversity. Microbial composition differed among subway surface types. (a) Taxa summary showing the most abundant genera. (b) Beta diversity at the genus level, Bray-Curtis based non-metric multidimensional scaling (NMDS) plot of surface types samples.

We sampled surfaces from turnstiles, escalator handrails, stair handrails, platform floors, poles, and train seats (Fig 1a). Relative abundances of surfaces microbiomes were higher for the phyla Proteobacteria (31 ± 8.6%), Actinobacteria (30 ± 9.2%), Firmicutes (24 ± 7.7%), Bacteroidetes (9.5 ± 4.5%), and Fusobacteria (1.5% ± 5.4%). At the genus level, the five most abundant taxa comprised 36% of the total abundance: *Acinetobacter* (10.5 ± 5.4%), *Corynebacterium* (8.2 ± 6.0%), *Streptococcus* (7.3% ± 4.2%), *Staphylococcus* (6.8% ± 5.2%), and *Cutibacterium* (3.4 ± 2.2%). Only nine genera were ubiquitous across all 40 samples (S8 Table), comprising 44% of the overall abundance. Fig 2 shows a summary of the genera composition. The five most abundant OTUs were *Acinetobacter sp*. (5.6 ± 3.6%), *Staphylococcus sp*. (3.8 ± 3.1%), *Cutibacterium acnes* (3.6 ± 5.6%), *Streptococcus sp*. (2.7 ± 1.2%), and *Staphylococcus epidermidis* (2.0 ± 1.2%) (Fig 2).

Alpha diversity varied among different surface types (for Shannon, Observed OTUs, Chao1, and Simpson metrics, *p* < 0.02, Kruskal-Wallis; S1 Fig). The platform floor was the most diverse surface, while the stair handrail and pole were the least diverse ones (*p* < 0.010, Dunn test; S1 Fig). Microbial composition differed among surface types (*p* = 0.001, F = 1.99, PERMANOVA), with different variance dispersion (*p* = 0.018, F = 3.35, PERMADISP2; S1 Fig).

Based on hierarchical clustering, the platform floor showed the most distinctive bacterial composition. Turnstiles, escalator handrails, and poles showed greater similarities (S1 Fig). Interestingly, stair handrail samples did not cluster with other hand-contact surfaces, although they displayed the highest variance dispersion, reflecting high heterogeneity among samples. In contrast, escalator handrails and turnstiles showed the lowest dispersion, indicating higher homogeneity among samples (Tukey’s HSD, *p-adjust* < 0.026, S1 Fig). No discriminant OTU was detected for stair handrail in comparison with other surfaces.

We also explored microbiome differences between regular and women-only wagons; we found no differences for any surface in terms of alpha or beta diversities (S2 Fig). Discriminant taxa analysis using the LEfSe algorithm did not show any overrepresented taxa between wagon types. Further analysis was performed using amplicon sequence variants (ASVs); also, no differences were detected (see below).

### Microbiome ecological succession after surface cleaning

We explored the changes in surface microbiomes after a cleaning event. We cleaned five poles with disinfectant towels and distilled water; the poles were sampled pre-cleaning (PC) and along five time-points post-cleaning (0, 0.5, 2, 8, and 48 h) (Fig 1b).

**Fig 3:**
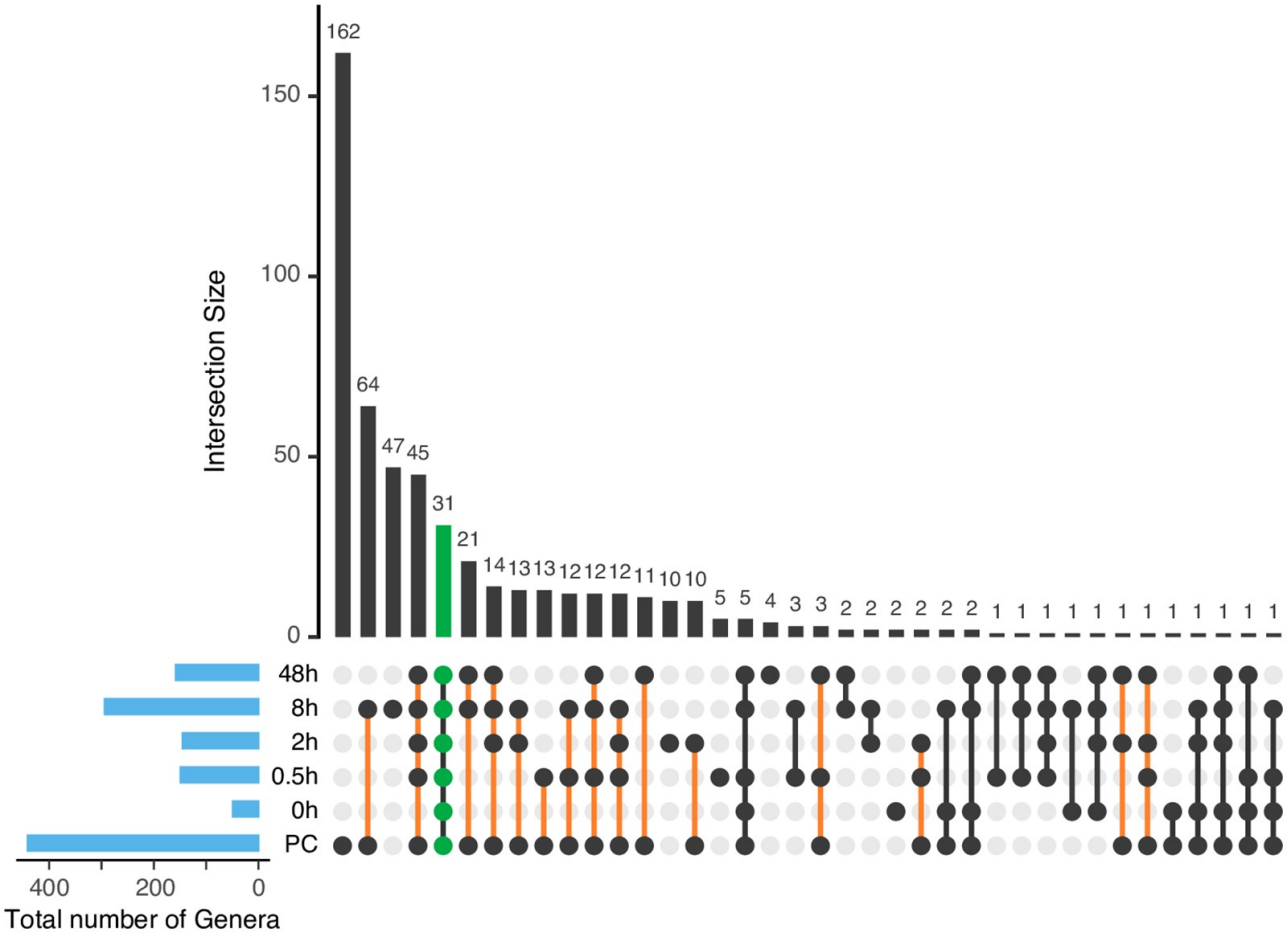
Unique and shared genera among time groups. Many genera are unique to each time group, and many resettled genera are not persistent. Upset plot of intersected genera among time points after cleaning; empty intersections are not shown. Taxa shared among all times are shown in green. Resettled taxa are shown with an orange line.

The cleaning procedure removed 96.38% (3,867 OTUs) of the initial OTUs. A total of 67 OTUs (31 genera) were shared among all-time groups, and they were not removed; these taxa included the most abundant genera. A total of 987 OTUs (234 genera) resettled the surfaces in at least one sample (Fig 3 and S3 Fig). Many of them were intermittently identified, and only 422 initial OTUs (148 genera) were detected in the 48-h samples. Within 30 min, 369 removed OTUs (100 genera) resettled on the pole surfaces. The pre-cleaning group showed the highest count of unique taxa (Fig 3 and S3 Fig), suggesting that rare taxa have not been completely established within 48 h. However, the high count of unique taxa in each time group, and the intermittent identification of taxa, suggest that rare taxa are not persistent.

**Fig 4:**
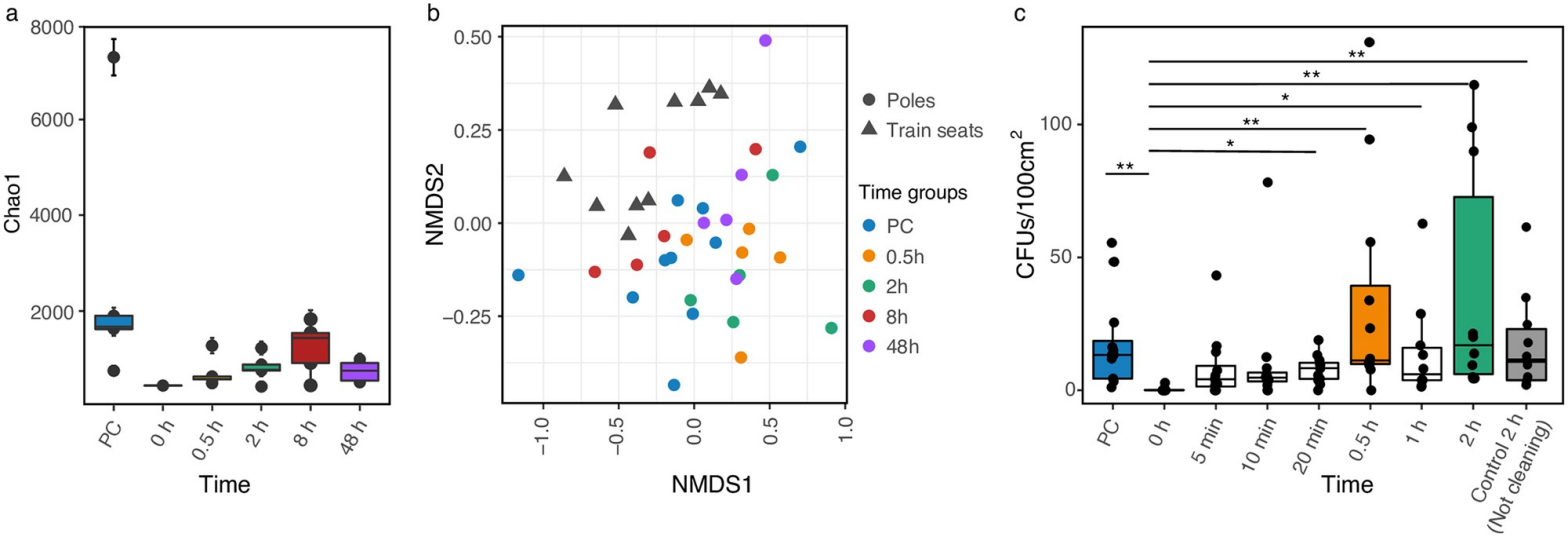
Pole microbiome diversity succession measured by 16S amplicons and colony-forming units (CFUs) after a cleaning event. (a) Alpha diversity and (b) beta diversity did not resettle within 48 h after cleaning. Nevertheless, the CFU count was regained within minutes (c). (a) Alpha diversity boxplots show the Chao1 richness estimator. (b) Non-metric multidimensional scaling (NMDS) ordination with Bray-Curtis dissimilarity at the genus level. (c) CFUs in seven time-points plus two controls: pre-cleaning (PC) and time control at two h (not cleaning). The last control pretends to evaluate the microbiome’s natural changes in real-time (*p < 0.05 and **p < 0.005, Dunn’s test).

Cleaning of poles significantly reduced sample biomass, hindering 16S gene amplification. We only obtained amplicons from one out of five samples for the first time-point, suggesting that the cleaning procedure was carried out properly. Richness comparison among time groups (not including 0 h, N = 1) yielded significant results (Chao1, *p* = 0.038, Kruskal-Wallis). However, pairwise comparison removed this significance (*p* > 0.05, Dunn’s test; Fig 4a). Beta diversity showed significant differences among group compositions (*p* = 0.001, F = 1.46, PERMANOVA; Fig 4b), while similar dispersion was observed (*p* = 0.867, F = 0.33, PERMADISP2). Set analysis suggests that after cleaning, pole microbiomes acquire a composition different from that of the pre-cleaning samples.

We also explored bacterial colonization dynamics for shorter periods (< 0.5 h) with a cultivation based-method. After the same cleaning procedure, 10–12 poles were sampled in seven-time points: 0 h, 5 min, 10 min, 20 min, 0.5 h, 1 h, and 2 h. Results showed that 5 minutes were sufficient to reach a similar number of colony-forming units (CFU) when compared to the pre-cleaning group (PC vs. 5 min, *p* > 0.050, Dunn’s test; Fig 4c).

### Passenger hand microbiome after traveling

**Fig 5:**
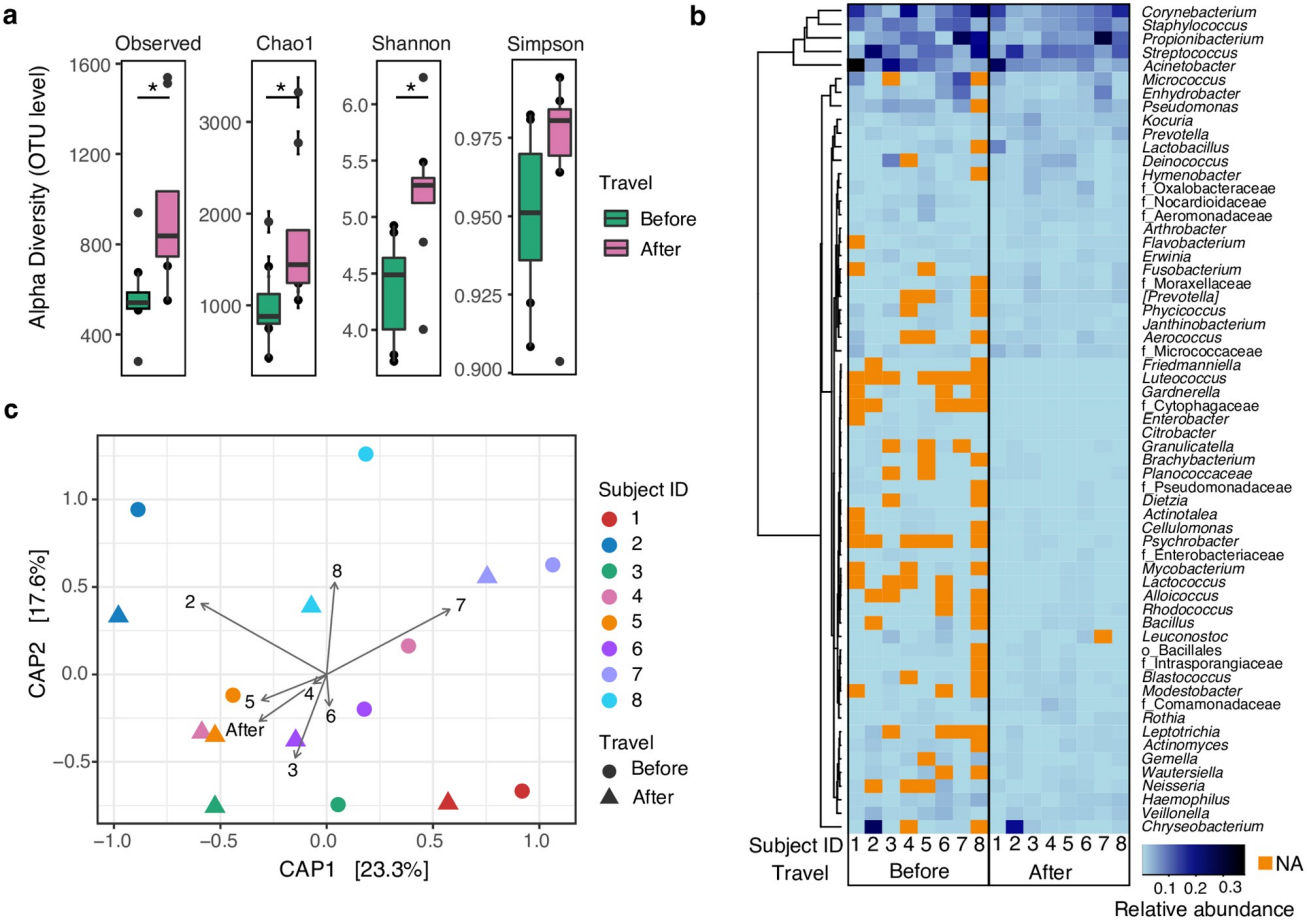
Changes in the passenger hand microbiome before and after traveling without handwashing. (a) Alpha diversity is increased after traveling (* *p* < 0.020). (b) Heatmap, using Manhattan distances, showing the relative abundance of all genera shared among subjects (columns, denoted by a number) before or after traveling. The subjects showed a closer microbiome profile after traveling. Letters before taxa indicate the best possible phylogenetic assignment (o: order and f: family). (c) Constrained analysis of principal coordinates (CAP) at the genus level, showing significant segregation for SubjectID and Travel (before and after) variables (*p* = 0.001, F = 2.4 and *p* = 0.006, F = 2.0, ANOVA-like permutation test for CAP).

Microbiome changes during a subway trip were measured in eight volunteers after an 11-station ride. Two procedures were evaluated: 1) traveling without previous handwashing (Fig 5) and 2) traveling with previous handwashing (S4 Fig). Handwashing was done using traditional soap and water protocol.

Subway traveling increased bacterial alpha diversity. Although, the handwashing immediately reduced biomass and diversity, traveling increased bacterial diversity to the same levels than after traveling without the handwashing procedure (OTU level: *p* < 0.020, for Observed, Chao1, and Shannon; Fig 5a and S4 Fig). The increased bacterial diversity may not be related to the touched surface type or the number of touched surfaces (*p* = 0.317, R^2^: 0.165; linear regression from net Shannon diversities and number of touched surfaces). Further exploration of the passenger-surface contact frequency and nature (intermittent or dragging-like) might elucidate this relationship.

After traveling, the observed OTUs increased by a mean count of 167.0% without (S2 Table) and 408.1% with the handwashing procedure (S3 Table). Unwashed hands lost 68.1% and washed hands 65.3% of their OTUs and acquired 135.1 and 254.4% of new OTUs. Unwashed hands conserved only 31.9% of the OTUs, while washed hands retained 34.7% of OTUs, including the most abundant ones. Additionally, small decreases of the most abundant taxa were observed after traveling with unwashed hands: *Acinetobacter* (11.7–7.7%), *Corynebacterium* (11.1–8.0%), *Streptococcus* (10.2–8.7%), *Cutibacterium* (9.4–7.8%), and *Staphylococcus* (6.9–5.9%) (S5 Fig). Similar changes were observed for most taxa for washed hands.

**Fig 6:**
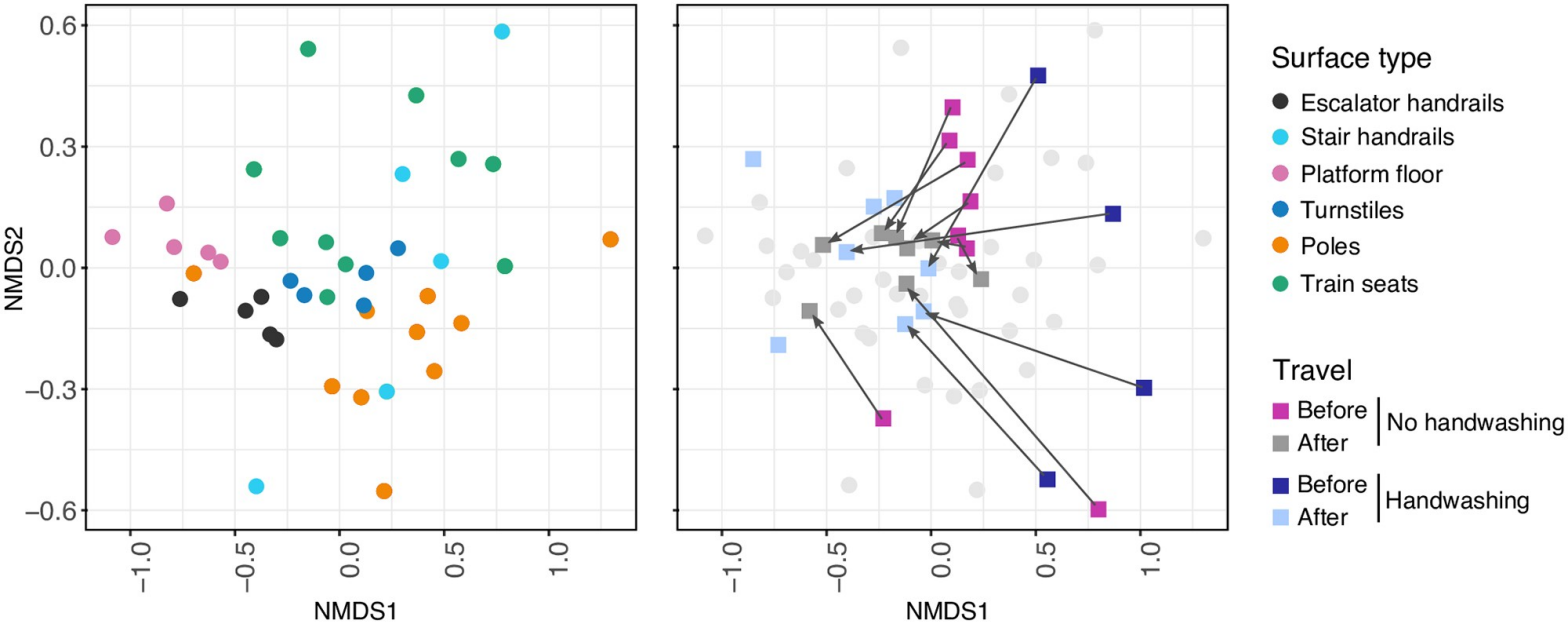
Hand microbiome composition converges after traveling. Non-multidimensional scaling (NMDS) ordination bi-plot performed with Bray Curtis distance for no handwashing and handwashing and different surface types, at the genus level. Arrows connect the same subjects from before to after traveling. Unpaired dots are samples without matching before or after travel comparison because of low metagenomic DNA yield (washed hands).

Subway passenger microbiome profiles converged after traveling. The number of taxa shared among the eight passengers increased after traveling without handwashing (Figs 5b and 6 and S4 Fig). Constrained analysis of principal coordinates (CAP) supported the microbial convergence after a subway ride, showing that the subject identifier variable (SubjectID) followed by the travel variable (before and after) significantly explained group segregation (*p* = 0.001, F = 2.4 and *p* = 0.006, F = 2.0, respectively; ANOVA-like permutation test for CAP, Fig 5c). The handwashing procedure analysis showed similar results (*p* = 0.003, F = 1.9), although the SubjectID variable reduced to marginal significance (*p* = 0.049, F = 1.3; S4 Fig). The convergence of the subject microbial composition after traveling can also be visualized in an NMDS ordination; SubjectIDs after traveling were closer to each other (Fig 6). Additionally, they became closer to the subway surface profiles, suggesting higher similarities with the surface microbiome, particularly evident for the handwashing group.

In summary, subway traveling increased hand microbial diversity and promoted passenger microbiome convergence. Although handwashing before traveling had an immediate effect on microbiome profiles, diversity and composition reached similar characteristics than after traveling without handwashing.

### ASVs analysis

We reanalyzed the central questions of this study using amplicon sequence variants (ASV). Both ASV and OTU are methodologies for data reduction. While ASVs are generated by parsing identical sequences and eliminating rare sequences, OTUs are generated by sequence clustering. Presenting the ASV approach may allow a more inclusive understanding of the data since it can provide strain resolution. Microbial diversity at the ASV level showed similar results than for the OTU analysis in alpha and beta diversity for subway surfaces and hands before and after traveling. Previous subway studies have reported the presence of *E*. *coli*, or even *Escherichia*, as opposed to this study. The ASV analysis detected 13 *Escherichia-Shigella* in some surface and passenger samples without any particular pattern. We compared regular and women-only wagons, identifying the presence and changes in the relative abundance of specific vaginal-associated bacteria based on the Silva database. This database includes a higher number of vaginal-associated bacteria than GreenGenes. Similar to the OTU analysis, no differences were found (S9 and S10 Tables and S6 Fig).

## Discussion

Microbial subway surfaces have been characterized previously in New York, Boston, Oslo, and México City [16–19]. Additionally, the hand microbiome has been explored for Hong Kong subway passengers [21]. This work complements the previous study of Mexico City by incorporating other surfaces into the research and analyzing women’s wagons. Furthermore, this work contributes to studying the world’s subway microbiomes by exploring the taxonomic contribution of subway surfaces to the passengers’ hands microbiome.

The Mexico City subway showed different microbial compositions among surface types. Differences in the kind of materials and the type of human body interaction may be shaping these profiles [17,19]. Although escalator handrails and poles are surfaces typically wrapped by the passengers’ hands, we found substantial differences in diversity and compositions among them. The higher porosity in the escalator rubber grips may provide a bigger contact surface and a higher faculty of harboring nutrient particles that facilitate bacterial growth. Additionally, it may serve as a humidity reservoir for bacteria [36]. On the contrary, poles are polished metal surfaces that reduce bacterial adherence and persistence. The platform floor, with the most distinctive composition, receives soil and dust particles carried in via shoes, while seats are impacted by the commuters’ clothes. Turnstiles, handrails, and poles show the microbial input of hands and clothes.

We also explored whether regular and women-only wagons displayed differentiated microbiomes, based on the previously reported sex-based microbial differences and building-occupiers microbial associations [4,5,9,10,37]. We did not find distinctions between regular and women-only wagons. Microbiome sex-based signals in trains may be hidden by the high intrinsic diversities of the subway surfaces. A female signal would probably require contact with a low, exposed body part (*e*.*g*., thighs or urogenital area) [9,38,39]. Additionally, these wagons are intermittently occupied by women. The first wagons are for women only, so each time a train reaches a terminal station, there is a railroad switch, reversing train wagon order. A 20-30-min ride would probably be not long enough to build a female microbial fingerprint. Additionally, a possible PCR primer bias might be limiting the detection of vaginal species (*e*.*g*., *Lactobacillus* spp.), which may be difficult accessing this comparison.

### The passenger microbial input

We described the poles as the least bacterial diverse surface and the most frequent passenger wrap surface (97%). Such a highly perturbed surface might be of particular interest in controlling microbial dispersal with health implications. In this study, we observed that pole cleaning effectively removed microbes. However, we found microbial resettling with few passenger interactions with bacterial richness and CFU counts similar to pre-cleaning levels. The fact that microbes rapidly resettle suggests that cleaning is effective short periods. Although the pole’s smoothness may avoid microbial accumulation, it might promote a high microbial exchange rate. In practical terms, the passenger microbes are rapidly wiped out by the next passenger.

We observed changes in the pole microbial compositions across time groups and did not detect an evident ecological succession sign. However, we cannot rule out a slow succession process. Gibbons et al. [38], have followed the microbial colonization on restroom floors and observed an early successional community composition within 8 h and a late-successional state over weeks to months. The observed high perturbation frequency of poles and the scarce deposition area of its material may impair a community structure’s development over time [40].

Further studies of cleaning porous surfaces such as escalator handrails (used by 86% of the passengers) may be essential to prevent microbial spreads; however, microbial removal efficiency must be tested. We also propose to pay particular attention to the cleaning of the floor, as floor surfaces are highly diverse, and the dust can be easily lifted with air and breathed in by passengers. Alternatively, self-cleaning building materials may be a long-term strategy for controlling bacterial colonization on surfaces [41].

### Passenger hand microbiome after a subway ride

We showed that the hands are the primary means of interaction with subway surfaces. Additionally, we observed that the face/head area was the area most commonly touched by users (73%). Interaction with mucosa was relatively high in prevalence (17%, 10-min trip). The mucosa has a particular health relevance, since reaching the nasal or conjunctival mucosa with the hands can lead to the transmission of diseases through the self-inoculation of microorganisms [42]. In contrast to the skin, with a robust mechanical barrier, the mucosa is an exposed area for external agents directly in contact with the immune system. Although recognition and protection against environmental agents are continually occurring in this area, the mucosa can also be vulnerable to disruption and microbial colonization. A persistent establishment would imply interactions with the immune system [43], while a transient establishment involves bacterial dispersal to other surfaces.

Bacterial adherence ability plays a vital role in the microbial exchange. It might be influenced by temperature and pressure conditions and the hydrophobic–hydrophilic properties of the interacting surfaces [44]. These properties may vary by surface characteristics and passenger hand microenvironment (pH, moisture, sebum level).

After a subway trip, the passenger hand microbiome increased in diversity. This increment is consistent with the higher diversity found on subway surfaces, which may be built by the contribution of several passengers and soil presence [19]. The hand from one person is a heterogeneous microbial source with high inter-individual variation [45]. We also detected commuter variation; only 7% of the genera were shared among passenger’s hands before traveling. This high inter-variation may be due to intrinsic factors such as age, sex, and extrinsic lifestyle-dependent factors: use of skincare products, pet ownership, allergies, alcohol consumption, time spent outdoors [10,37,39].

We showed that hand microbial composition converged among passengers after traveling. This convergence means that one trip is enough to perceive the effect of building cohabitation [5,9,37]. Changes in the hand microbiome were expected, as hands were constantly interacting with different surfaces. Hands show higher temporal variability than other body sites (reviewed in [43]).

Besides diversity changes due to travel, we observed microbial fingerprint preservation within passengers. Highly abundant taxa persist within subjects [24], while transient bacteria are the primary variability source [39]. Microbial fingerprint has also been observed in volunteers sampled and resampled at 4 to 6 months later, with no significant change over time [46].

Under normal circumstances and for healthy people, it does not seem to be a risk exposing yourself to a subway ride. Furthermore, it could even be argued that it is a way to increase microbial diversity and improve your immune system [19]. However, for immunosuppressed people or in a pandemic, we recommend washing your hands as soon as you reach the destination and trying not to touch your face during the trip. Other subway studies that include air and virus analysis are relevant to continue understanding the implications of traveling by subway.

## Conclusions

We detected the effect of a trip on passengers of the Mexico City subway. Each time a passenger travels in the subway, she or he leaves some bacteria and brings others. Passengers become similar to the subway surfaces, and they are more alike among each other after traveling. Each passenger’s microbial fingerprint is preserved, mostly explained by high-abundance taxa. Although most bacteria will not persist, traveling in the subway is a way of sharing our microbes.

Poles are the most touched surface in the Mexico City subway. Pole cleaning reduces microbial richness and diversity. However, the amount of CFU is quickly restored within 5 min after pole cleaning. Even so, the microbial composition is not resettled within 48 hours. We think that the lack of restoration of the initial microbial community is due to the variability of the hosts’ microbiomes and the lack of rare taxa persistence.

## Supporting information

S1 Fig Subway surface microbiome diversity in stations and regular train wagons (N = 5 samples per surface type). The platform floor was most diverse, with the most distinctive composition. (a) Alpha diversity measures for surface type at the OTU level. Pairwise comparison showed that platform floor diversity was higher than that of stair handrails and poles (* *p* < 0.01, Nemenyi-tests). (b) Hierarchical clustering of individual surface samples, colored by surface type. Hierarchical clustering analyses were performed with the ward.2 method and Bray Curtis dissimilarity. (c) Variance dispersion among surface types (* *p* < 0.02, PERMADISP2; *p*.*adjust* < 0.026, Tukey´s HSD). Distances to centroid groups were calculated by reducing the original Bray Curtis dissimilarity to principal coordinates.
(PDF)

S2 Fig There are no microbiome differences between regular and women-only train wagons at the OTU level (N = 5 samples per category). (a) Non-metric multidimensional scaling (NMDS) ordination with Bray dissimilarity showing spatial distribution of sample groups (poles, *p* > 0.18, F = 1.13; train seats, *p* > 0.59, F = 0.95, PERMANOVA; NMDS stress = 0.20). (b) Alpha diversity measures at the OTU level. No significance was found between groups for any measure (*p* > 0.5, Kruskal-Wallis).
(PDF)

S3 Fig Unique and shared OTUs among time groups. Many OTUs are unique to each time group, and many resettled taxa are not persistent (a) Upset plot of intersected OTUs among time points after cleaning; empty intersections are not shown. Taxa shared among all times are shown in green. Resettled taxa are indicated with an orange line.
(PDF)

S4 Fig Changes in the passenger hands microbiome before and after traveling with handwashing. (a) Alpha diversity is increased (* *p* < 0.020). (b) Heatmap showing the relative abundance of all common taxa at the genus level, before or after traveling per SubjectID, denoted by a number. Row dendrogram arrangement based in Manhattan distance. (c) Constrained analysis of principal coordinates (CAP) at the genus level, showing significant segregation of SubjectID and Travel variables (*p* = 0.049, F = 1.3 and *p* = 0.003, respectively, F = 1.9, ANOVA-like permutation test for CAP).
(PDF)

S5 Fig Taxa summary at the genus level per subject id. Each subject fingerprint is preserved after traveling (a) Before and after traveling without handwashing. (b) With handwashing. Missing bars come from samples not sequenced due to low DNA biomass.
(PDF)

S6 Fig Taxa summary at genus level from ASVs generated with DADA2 and taxonomy assign based on Silva database. Similar to the OTU analysis, the most abundant phyla were Proteobacteria (37%), Firmicutes (24%), Actinobacteria (20%), and Bacteroidetes (9.3%). However, Cyanobacteria (3.3%, no chloroplast) appeared in the fifth position. The most abundant ASVs were *Acinetobacter lwoffii* (0.72%), *Streptococcus* sp. (0.68%), *Streptococcus* sp. (0.59%), *Acinetobacter lwoffii* (0.56%), and *Propionibacterium acnes* (0.56%). A total of 17 ASVs named as archaea were identified (*Methanobrevibacter*, *Candidatus Nitrososphaera SCA1170*, *Candidatus Nitrososphaera SCA1145*, *Methanosaeta vadinCA11*, *Methanosaeta*, *Natronococcus*, *Methanobacterium*, *Halococcus*, among other not identified genera).
(PDF)

S1 Table Number of samples collected and successfully processed. (PDF)

S2 Table Number of OTUs kept, lost, and newly acquired after one subway travel per passenger without handwashing. (PDF)

S3 Table Number of OTUs kept, lost, and newly acquired after one subway travel per passenger with handwashing. (PDF)

S4 Table Frequency and prevalence of activities observed in passengers during a train trip. (PDF)

S5 Table Percentage of passengers touching the handrails of escalators and stairs. (PDF)

S6 Table Relative humidity, temperature, collection date, DNA concentration, and the number of raw sequences per sample. (PDF)

S7 Table Numbers of raw reads, paired-end reads, and OTUs. (PDF)

S8 Table Shared genera among samples within the same surface type and among all samples. (PDF)

S9 Table Summary of sequences and ASVs in 89 samples. (PDF)

S10 Table Number of vaginal-associated taxa as a female environmental indicator. (PDF)
