# Supplementary figures and images for: Passenger-surface microbiome interactions in the subway of Mexico City
Source: PLoS One. 2020 Aug 19;15(8):e0237272. doi: 10.1371/journal.pone.0237272 (PMC7437895; doi:10.1371/journal.pone.0237272)

**a**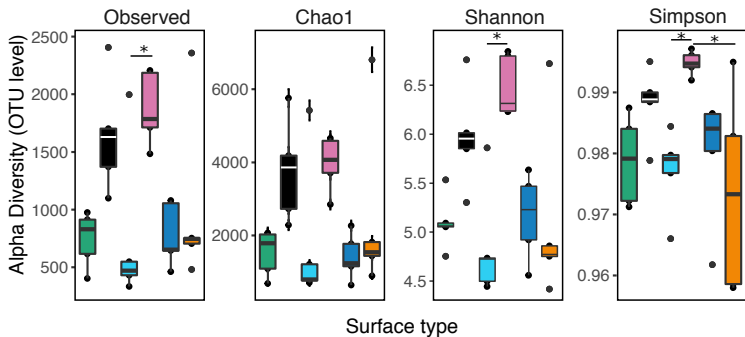**b**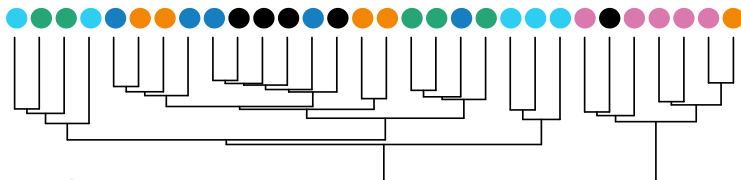**c**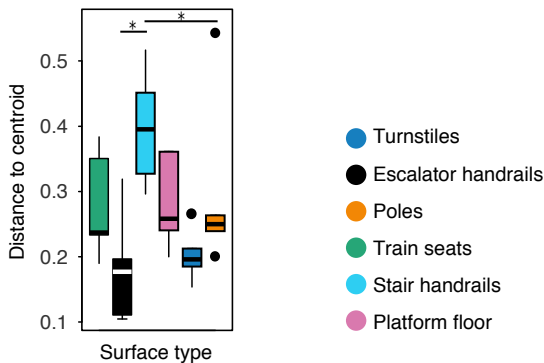

Supplement: S1 Fig — The platform floor was most diverse, with the most distinctive composition. (a) Alpha diversity measures for surface type at the OTU level. Pairwise comparison showed that platform floor diversity was higher than that of stair handrails and poles (* p < 0.01, Nemenyi-tests). (b) Hierarchical clustering of individual surface samples, colored by surface type. Hierarchical clustering analyses were performed with the ward.2 method and Bray Curtis dissimilarity. (c) Variance dispersion among surface types (* p < 0.02, PERMADISP2; p.adjust < 0.026, Tukey´s HSD). Distances to centroid groups were calculated by reducing the original Bray Curtis dissimilarity to principal coordinates. (PDF) [file pone.0237272.s001.pdf]

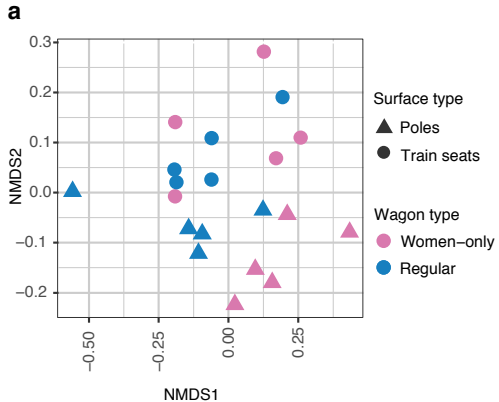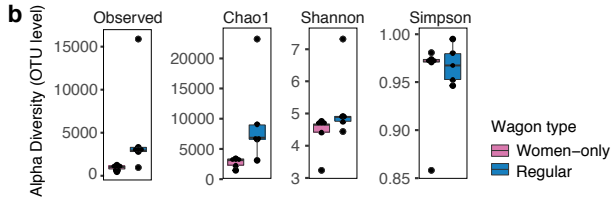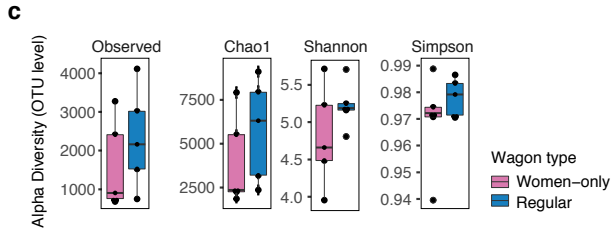

Supplement: S2 Fig — (a) Non-metric multidimensional scaling (NMDS) ordination with Bray dissimilarity showing spatial distribution of sample groups (poles, p > 0.18, F = 1.13; train seats, p > 0.59, F = 0.95, PERMANOVA; NMDS stress = 0.20). (b) Alpha diversity measures at the OTU level. No significance was found between groups for any measure (p > 0.5, Kruskal-Wallis). (PDF) [file pone.0237272.s002.pdf]

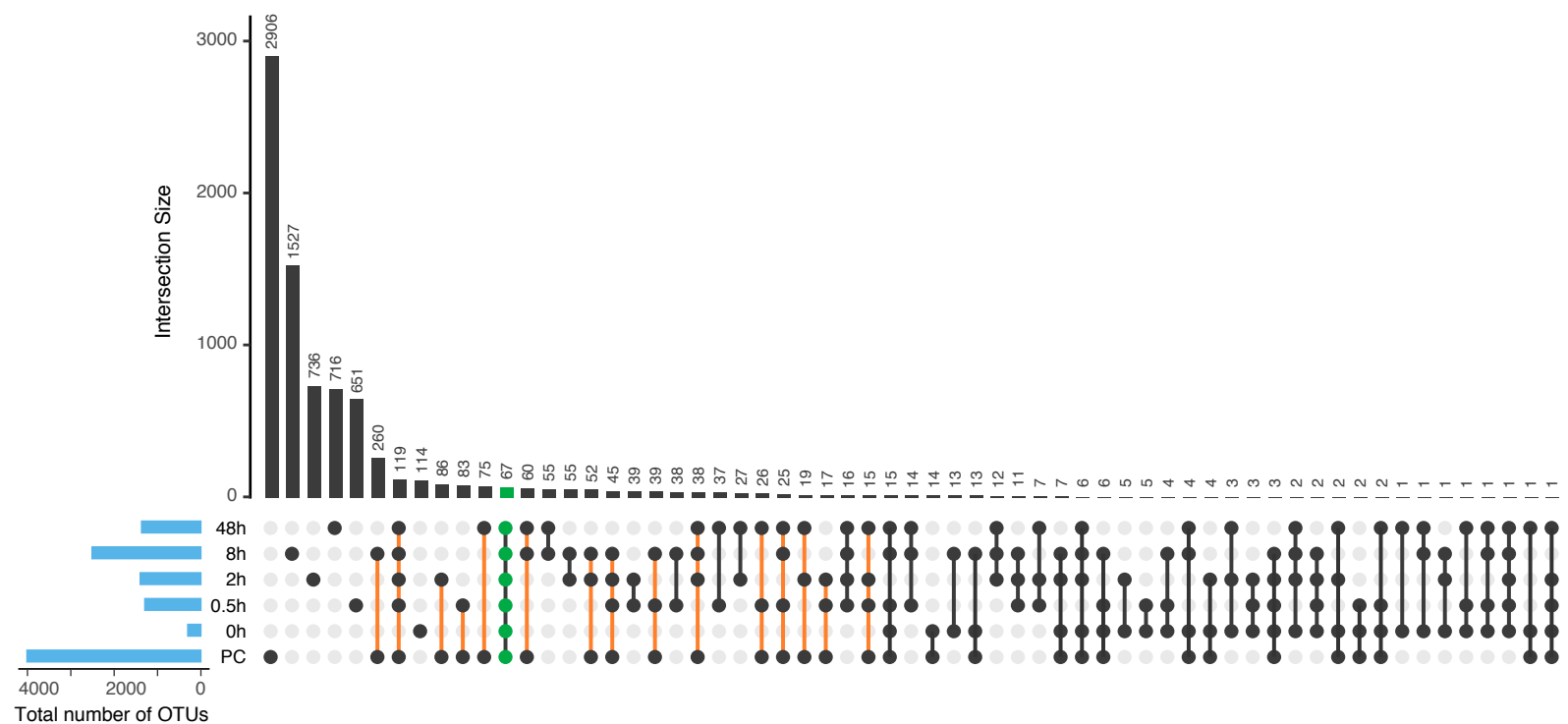

Supplement: S3 Fig — Many OTUs are unique to each time group, and many resettled taxa are not persistent (a) Upset plot of intersected OTUs among time points after cleaning; empty intersections are not shown. Taxa shared among all times are shown in green. Resettled taxa are indicated with an orange line. (PDF) [file pone.0237272.s003.pdf]

**a**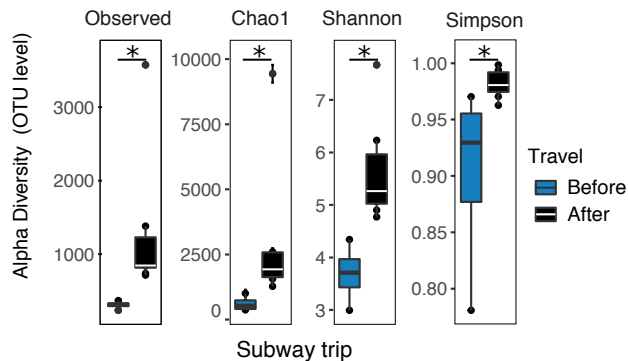**b**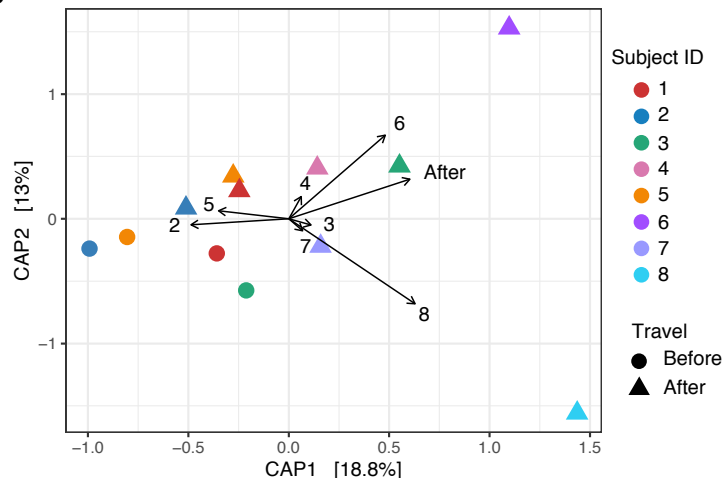**c**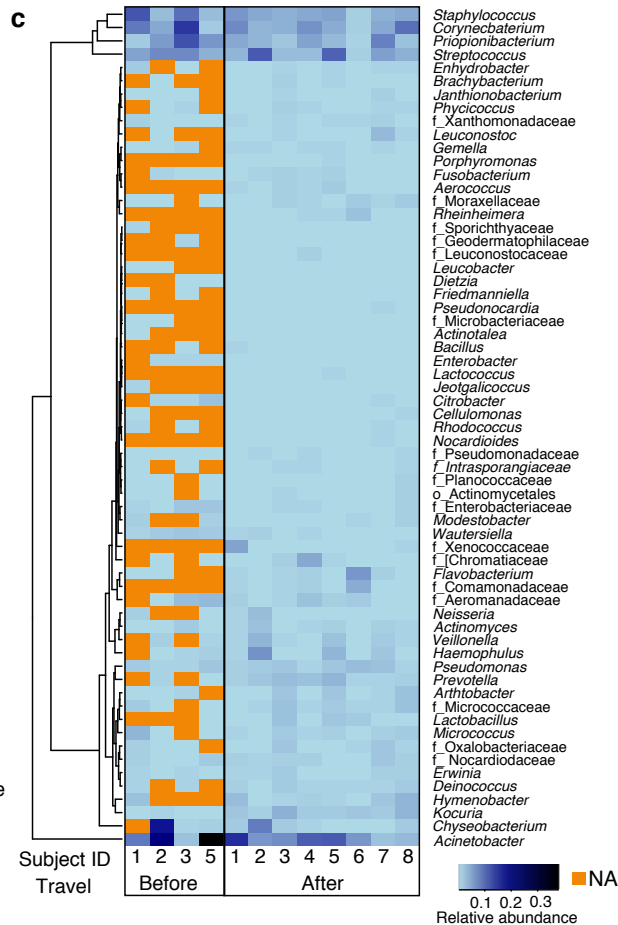

Supplement: S4 Fig — (a) Alpha diversity is increased (* p < 0.020). (b) Heatmap showing the relative abundance of all common taxa at the genus level, before or after traveling per SubjectID, denoted by a number. Row dendrogram arrangement based in Manhattan distance. (c) Constrained analysis of principal coordinates (CAP) at the genus level, showing significant segregation of SubjectID and Travel variables (p = 0.049, F = 1.3 and p = 0.003, respectively, F = 1.9, ANOVA-like permutation test for CAP). (PDF) [file pone.0237272.s004.pdf]

a

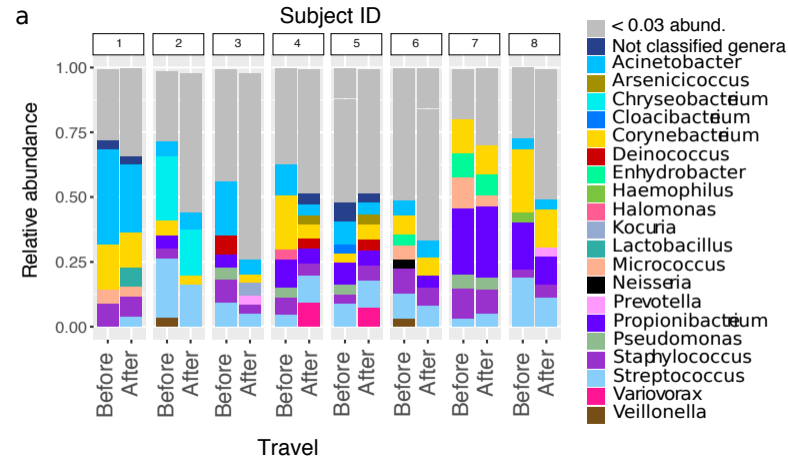

b

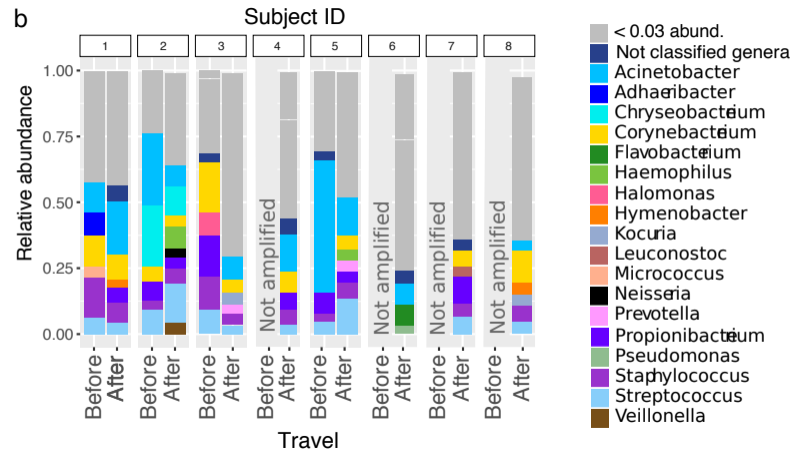

Supplement: S5 Fig — Each subject fingerprint is preserved after traveling (a) Before and after traveling without handwashing. (b) With handwashing. Missing bars come from samples not sequenced due to low DNA biomass. (PDF) [file pone.0237272.s005.pdf]

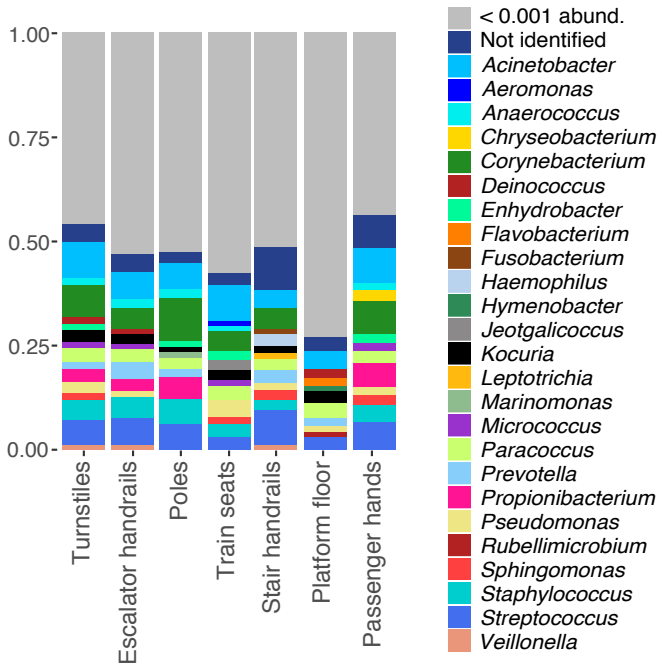

Supplement: S6 Fig — Similar to the OTU analysis, the most abundant phyla were Proteobacteria (37%), Firmicutes (24%), Actinobacteria (20%), and Bacteroidetes (9.3%). However, Cyanobacteria (3.3%, no chloroplast) appeared in the fifth position. The most abundant ASVs were Acinetobacter lwoffii (0.72%), Streptococcus sp. (0.68%), Streptococcus sp. (0.59%), Acinetobacter lwoffii (0.56%), and Propionibacterium acnes (0.56%). A total of 17 ASVs named as archaea were identified (Methanobrevibacter, Candidatus Nitrososphaera SCA1170, Candidatus Nitrososphaera SCA1145, Methanosaeta vadinCA11, Methanosaeta, Natronococcus, Methanobacterium, Halococcus, among other not identified genera). (PDF) [file pone.0237272.s006.pdf]
