# Supplementary material for: Passenger-surface microbiome interactions in the subway of Mexico City
Source: PLoS One. 2020 Aug 19;15(8):e0237272. doi: 10.1371/journal.pone.0237272 (PMC7437895; doi:10.1371/journal.pone.0237272)
Supplement: S1 Table — (PDF) [file pone.0237272.s007.pdf]

Table S1. Number of samples collected and successfully processed.

	N of samples	
**Sample type**	**Sampled**	**Successfully processed**
**Turnstiles**	**5**	**5**
**Stairs handrails**	**5**	**5**
**Escalator handrails**	**5**	**5**
**Platform floors**	**5**	**5**
**Train seats**		
Women-only wagon	5	5
Regular wagon	5	5
**Poles**		
Women-only wagon	5	5
P0h (Pre cleaning)	5	5
0h (Post cleaning)	5	1
0.5h (Post cleaning)	5	5
2h (Post cleaning)	5	5
8h (Post cleaning)	5	5
48h (Post cleaning)	5	5
**Passenger hands**		
Before travelling	8	8
After travelling	8	8
Before travelling (Hand washing)	8	4
After travelling (Hand washing)	8	8
**Total**	**97**	**89**
