# Supplementary material for: Passenger-surface microbiome interactions in the subway of Mexico City
Source: PLoS One. 2020 Aug 19;15(8):e0237272. doi: 10.1371/journal.pone.0237272 (PMC7437895; doi:10.1371/journal.pone.0237272)
Supplement: S2 Table — (PDF) [file pone.0237272.s008.pdf]

Table S2. Number of OTUs kept, lost, and newly acquired after one subway travel per pessenge without handwashing.

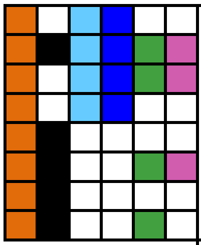

Touched surface: Poles	Escalator handrails	Stair handrails	Turnstiles	Train seats	Platform floors	Subject ID	N of OTUs: Before	After	Kept	Lost	Newly adquired	Shannon: Before	After
						**1**	675	759	190	485	569	4.3	4.9
						**2**	940	1539	326	614	1213	4.6	5.8
						**3**	516	1512	197	319	1315	4.6	6.5
						**4**	508	864	159	349	705	4.7	5.4
						**5**	535	809	162	373	647	5.0	5.4
						**6**	547	704	162	385	542	4.9	5.4
						**7**	556	551	148	408	403	3.8	4.1
						**8**	281	875	110	171	765	3.8	5.4
**Touched surface**						**mean**	**569.8**	**951.6**	**181.8**	**388.0**	**769.9**	**4.5**	**5.4**
						**sd**	**185.2**	**368.8**	**64.0**	**127.9**	**325.1**	**0.5**	**0.7**
