# Supplementary material for: Passenger-surface microbiome interactions in the subway of Mexico City
Source: PLoS One. 2020 Aug 19;15(8):e0237272. doi: 10.1371/journal.pone.0237272 (PMC7437895; doi:10.1371/journal.pone.0237272)
Supplement: S3 Table — (PDF) [file pone.0237272.s009.pdf]

Table S3. Number of OTUs kept, lost, and newly acquired after one subway travel per pessenge with handwashing.

Touched surface														
Poles	Escalator handrails	Stair handrails	Turnstiles	Train seats	Platform floors	Subject ID	N of OTUs					Shannon	
							Before	After	Kept	Lost	Newly adquired	Before	After
■		■	■			**1**	319	715	114	205	601	4.4	5.1
■	■	■	■	■	■	**2**	370	848	131	239	717	3.7	5.0
■		■	■	■	■	**3**	237	1179	93	144	1086	3.9	6.2
■		■	■			**4**	NA	841	NA	NA	NA	NA	5.5
■	■					**5**	314	843	92	222	751	3.1	5.2
■	■			■	■	**6**	NA	1381	NA	NA	NA	NA	6.5
■	■					**7**	NA	739	NA	NA	NA	NA	5.4
■	■			■		**8**	NA	3574	NA	NA	NA	NA	8.5
Touched surface						**mean**	**310.0**	**1265.0**	**107.5**	**202.5**	**788.8**	**3.7**	**5.9**
						**sd**	**54.9**	**960.9**	**18.7**	**41.4**	**208.3**	**0.5**	**1.2**
