# Supplementary material for: Passenger-surface microbiome interactions in the subway of Mexico City
Source: PLoS One. 2020 Aug 19;15(8):e0237272. doi: 10.1371/journal.pone.0237272 (PMC7437895; doi:10.1371/journal.pone.0237272)
Supplement: S4 Table — (PDF) [file pone.0237272.s010.pdf]

**Table S4. Frequency and prevalence of activities observed in passengers duringa a train trip.**

| Activity | All (N=120) | | | Adults (20-65 years) (N=67) | | | Elders (>65 years) (N=53) | | | | |
|---|---|---|---|---|---|---|---|---|---|---|---|
| | N of contacts per passenger/10 min | | % of passengers | N of contacts per passenger/10 min | | % of passengers | N of contacts per passenger/10 min | | % of passengers | *p* value (Wilcoxon test) | |
| | Median | Mean | | Median | Mean | | Median | Mean | | | |
| **Touching surfaces/objects with the hand** | | | | | | | | | | | |
| Any wagon surface | 4.06 | 4.86 | 91.7 | 3.53 | 4.39 | 88.1 | 5.00 | 5.46 | 96.2 | *0.030* | * |
| *Pole* | 3.49 | 4.44 | 89.2 | 2.73 | 4.08 | 86.6 | 4.44 | 4.90 | 92.5 | *0.051* | |
| *Door* | 0.00 | 0.30 | 10.8 | 0.00 | 0.24 | 11.9 | 0.00 | 0.37 | 9.4 | *0.761* | |
| *Seat* | 0.00 | 0.12 | 7.5 | 0.00 | 0.06 | 4.5 | 0.00 | 0.19 | 11.3 | *0.148* | |
| *Roof* | 0.00 | 0.01 | 0.8 | 0.00 | 0.01 | 1.5 | NA | NA | 0.0 | *NA* | |
| Face/head area | 2.22 | 3.94 | 73.3 | 2.58 | 3.67 | 79.1 | 1.43 | 4.29 | 66.0 | *0.408* | |
| *Face skin* | 1.34 | 2.96 | 67.5 | 1.82 | 2.79 | 71.6 | 1.11 | 3.18 | 62.3 | *0.798* | |
| *Hair or scalp* | 0.00 | 0.70 | 26.7 | 0.00 | 0.62 | 32.8 | 0.00 | 0.81 | 18.9 | *0.148* | |
| *Face mucosa* | 0.00 | 0.24 | 16.7 | 0.00 | 0.24 | 19.4 | 0.00 | 0.24 | 13.2 | *0.439* | |
| *Ear canal* | 0.00 | 0.04 | 4.2 | 0.00 | 0.03 | 1.5 | 0.00 | 0.07 | 7.5 | *0.109* | |
| Personal article | 1.97 | 2.91 | 72.5 | 1.67 | 2.84 | 76.1 | 2.31 | 3.01 | 67.9 | *0.843* | |
| *Cellphone* | 0.00 | 0.54 | 30.0 | 0.00 | 0.92 | 49.3 | 0.00 | 0.05 | 5.7 | $3x10^{-7}$ | * |
| *Glasses* | 0.00 | 0.11 | 8.3 | 0.00 | 0.10 | 7.5 | 0.00 | 0.13 | 9.4 | *0.704* | |
| *Clothing* | 0.00 | 0.74 | 33.3 | 0.00 | 0.57 | 28.4 | 0.00 | 0.96 | 39.6 | *0.155* | |
| *Other (1)* | 0.72 | 1.52 | 52.5 | 0.25 | 1.25 | 50.7 | 0.91 | 1.87 | 54.7 | *0.471* | |
| Food consumption | 0.00 | 0.14 | 5.8 | 0.00 | 0.17 | 7.5 | 0.00 | 0.10 | 3.8 | *0.590* | |
| *Beverage* | 0.00 | 0.10 | 3.3 | 0.00 | 0.16 | 4.5 | 0.00 | 0.04 | 1.9 | *0.434* | |
| *With no hand contact* | 0.00 | 0.07 | 1.7 | 0.00 | 0.07 | 1.5 | 0.00 | 0.06 | 1.9 | *0.886* | |
| *With bare hands* | 0.00 | 0.01 | 0.8 | 0.00 | 0.02 | 1.5 | NA | NA | 0.0 | *NA* | |
| Another person | 0.00 | 0.14 | 5.8 | 0.00 | 0.05 | 1.5 | 0.00 | 0.26 | 11.3 | *0.024* | * |
| **Other activities** | | | | | | | | | | | |
| Money interchange (2) | 0.00 | 0.11 | 3.3 | 0.00 | 0.03 | 3.0 | 0.00 | 0.21 | 3.8 | *0.792* | |
| Sitting | 0.00 | 0.68 | 33.3 | 0.00 | 0.42 | 20.9 | 0.00 | 1.01 | 49.1 | *0.001* | * |
| Laying body on surface | 0.00 | 0.19 | 50.8 | 0.00 | 0.21 | 44.8 | 0.00 | 0.15 | 58.5 | *0.049* | * |
| Cough | 0.00 | 0.07 | 5.8 | 0.00 | 0.06 | 6.0 | 0.00 | 0.09 | 5.7 | *0.764* | |

(1) Bag, plastic bags, purse, book, newspaper, makeup, etc. (not including entering with a bag but having conta

(2) Buying or giving charity

* p value ≤0.05
