# Supplementary material for: Passenger-surface microbiome interactions in the subway of Mexico City
Source: PLoS One. 2020 Aug 19;15(8):e0237272. doi: 10.1371/journal.pone.0237272 (PMC7437895; doi:10.1371/journal.pone.0237272)
Supplement: S5 Table — (PDF) [file pone.0237272.s011.pdf]

Table S5. Percentage of passengers touching the handrails of escalators and stairs.

**Handrails**		**Passenger touching handrails, % (n/N)**
Escalators		86.2 (2283/2650)
Stairs		20.3 (973/4806)
	Up	*17.1 (425/2479)*
	Down	*23.6 (548/2327)*
