# Supplementary material for: Passenger-surface microbiome interactions in the subway of Mexico City
Source: PLoS One. 2020 Aug 19;15(8):e0237272. doi: 10.1371/journal.pone.0237272 (PMC7437895; doi:10.1371/journal.pone.0237272)
Supplement: S6 Table — (PDF) [file pone.0237272.s012.pdf]

**Table S6. Mesuread variables (relative humidity and temperature), collection date, DNA concentration, and number of raw sequences per saple.**

| sample_ID | sample_type | rel_air_humidity | air_temp | collection_date | DNA_UV (ng_uL) | raw_assembled_sequences |
|---|---|---|---|---|---|---|
| LA5 | skin_right_hand | 44 | 19 | 22-Nov-2018 | 5.08 | 33893 |
| LD5 | skin_right_hand | 38 | 22 | 22-Nov-2018 | 14.74 | 60172 |
| SD5 | skin_right_hand | 45 | 19 | 22-Nov-2018 | 86.29 | 40185 |
| SA5 | skin_right_hand | 40 | 22 | 22-Nov-2018 | 68.39 | 38808 |
| LA2 | skin_right_hand | 44 | 19 | 22-Nov-2018 | 1.27 | 77430 |
| LD2 | skin_right_hand | 38 | 22 | 22-Nov-2018 | 4.84 | 131270 |
| SA2 | skin_right_hand | 40 | 22 | 22-Nov-2018 | 8.74 | 48905 |
| SD2 | skin_right_hand | 45 | 19 | 22-Nov-2018 | 15.27 | 54193 |
| LD3 | skin_right_hand | 38 | 22 | 22-Nov-2018 | 19.09 | 198903 |
| SD3 | skin_right_hand | 45 | 19 | 22-Nov-2018 | 21.56 | 54015 |
| LA3 | skin_right_hand | 44 | 19 | 22-Nov-2018 | 31.57 | 91923 |
| SA3 | skin_right_hand | 40 | 22 | 22-Nov-2018 | 1.55 | 33912 |
| LD1 | skin_right_hand | 38 | 22 | 22-Nov-2018 | 2.09 | 87439 |
| SA1 | skin_right_hand | 40 | 22 | 22-Nov-2018 | 4.78 | 44297 |
| LA1 | skin_right_hand | 44 | 19 | 22-Nov-2018 | 1.07 | 15136 |
| SD1 | skin_right_hand | 45 | 19 | 22-Nov-2018 | 126.42 | 34589 |
| LD6 | skin_right_hand | 38 | 22 | 22-Nov-2018 | 23.57 | 77792 |
| SD6 | skin_right_hand | 45 | 19 | 22-Nov-2018 | 1.57 | 42246 |
| SA6 | skin_right_hand | 40 | 22 | 22-Nov-2018 | 10.39 | 33103 |
| LD4 | skin_right_hand | 38 | 22 | 22-Nov-2018 | 74.66 | 86562 |
| SD4 | skin_right_hand | 45 | 19 | 22-Nov-2018 | 22.07 | 54146 |
| SA4 | skin_right_hand | 40 | 22 | 22-Nov-2018 | 45.90 | 34314 |
| LD7 | skin_right_hand | 38 | 22 | 22-Nov-2018 | 112.31 | 44363 |
| SA7 | skin_right_hand | 40 | 22 | 22-Nov-2018 | 2.97 | 30958 |
| SD7 | skin_right_hand | 45 | 19 | 22-Nov-2018 | 28.73 | 36741 |
| LD8 | skin_right_hand | 38 | 22 | 22-Nov-2018 | 206.29 | 101256 |
| SA8 | skin_right_hand | 40 | 22 | 22-Nov-2018 | 4.40 | 19890 |
| SD8 | skin_right_hand | 45 | 19 | 22-Nov-2018 | 3.77 | 44467 |
| TP1 | turnstile | 47 | 22 | 29-Oct-2018 | 55.84 | 43941 |
| TP2 | turnstile | 48 | 21 | 29-Oct-2018 | 26.90 | 23529 |
| TP3 | turnstile | 49 | 21 | 29-Oct-2018 | 68.89 | 39309 |
| TP4 | turnstile | 52 | 20 | 29-Oct-2018 | 38.60 | 39106 |
| TP5 | turnstile | 52 | 20 | 29-Oct-2018 | 5.43 | 54493 |
| PR3 | stairs_handrail | 31 | 26 | 20-Nov-2018 | 50.89 | 121669 |
| PR1 | stairs_handrail | 34 | 24 | 20-Nov-2018 | 34.96 | 35905 |
| PR5 | stairs_handrail | 31 | 28 | 20-Nov-2018 | 98.64 | 53191 |
| PR4 | stairs_handrail | 36 | 27 | 20-Nov-2018 | 1.49 | 62671 |
| PR2 | stairs_handrail | 38 | 24 | 20-Nov-2018 | 2.44 | 48445 |
| PM3 | scalator_handrail | 33 | 26 | 20-Nov-2018 | 11.35 | 82668 |
| PM1 | scalator_handrail | 34 | 24 | 20-Nov-2018 | 18.41 | 71606 |
| PM5 | scalator_handrail | 31 | 28 | 20-Nov-2018 | 13.06 | 64366 |
| PM4 | scalator_handrail | 36 | 27 | 20-Nov-2018 | 28.04 | 79962 |
| PM2 | scalator_handrail | 38 | 24 | 20-Nov-2018 | 33.05 | 87440 |
| BF1 | pole | 44 | 27 | 31-Oct-2018 | 1.80 | 49284 |
| BF2 | pole | 43 | 27 | 31-Oct-2018 | 1.80 | 24771 |
| BF3 | pole | 44 | 27 | 31-Oct-2018 | 24.17 | 41976 |
| BF5 | pole | 46 | 27 | 31-Oct-2018 | 1.57 | 50129 |

| | | | | | | |
|---|---|---|---|---|---|---|
| B22 | pole | 49 | 28 | 29-Oct-2018 | 59.81 | 38287 |
| B23 | pole | 39 | 32 | 29-Oct-2018 | 86.71 | 41361 |
| B24 | pole | 37 | 32 | 29-Oct-2018 | 1.72 | 33335 |
| B25 | pole | 37 | 32 | 29-Oct-2018 | 20.59 | 76069 |
| Bc1 | pole | 56 | 20 | 29-Oct-2018 | 2.36 | 51959 |
| Bc2 | pole | 46 | 28 | 29-Oct-2018 | 7.40 | 24060 |
| Bc4 | pole | 46 | 29 | 29-Oct-2018 | 2.02 | 28946 |
| B41 | pole | 46 | 26 | 31-Oct-2018 | 1.83 | 45469 |
| B42 | pole | 46 | 26 | 31-Oct-2018 | 1.74 | 42237 |
| B45 | pole | 46 | 27 | 31-Oct-2018 | 1.14 | 72208 |
| B81 | pole | 58 | 25 | 29-Oct-2018 | 7.22 | 92576 |
| B82 | pole | 48 | 26 | 29-Oct-2018 | 3.15 | 41792 |
| B83 | pole | 48 | 27 | 29-Oct-2018 | 1.17 | 28386 |
| B84 | pole | 55 | 27 | 29-Oct-2018 | 1.87 | 36519 |
| B85 | pole | 47 | 29 | 29-Oct-2018 | 2.33 | 118089 |
| BP1 | pole | 56 | 20 | 29-Oct-2018 | 39.34 | 101097 |
| BP2 | pole | 46 | 28 | 29-Oct-2018 | 14.00 | 72347 |
| BP3 | pole | 50 | 29 | 29-Oct-2018 | 38.87 | 244572 |
| BP4 | pole | 46 | 29 | 29-Oct-2018 | 5.30 | 86486 |
| BF4 | pole | 47 | 27 | 31-Oct-2018 | 65.70 | 24143 |
| B21 | pole | 40 | 29 | 29-Oct-2018 | 21.78 | 18315 |
| Bc3 | pole | 50 | 29 | 29-Oct-2018 | 5.15 | 27565 |
| Bc5 | pole | 46 | 29 | 29-Oct-2018 | 19.36 | 25836 |
| B43 | pole | 48 | 26 | 31-Oct-2018 | 10.13 | 41416 |
| B44 | pole | 45 | 27 | 31-Oct-2018 | 22.41 | 21524 |
| BP5 | pole | 46 | 29 | 29-Oct-2018 | 96.23 | 29618 |
| B04 | pole | 46 | 29 | 29-Oct-2018 | 1.84 | 20708 |
| Pi2 | floor | 43 | 29 | 31-Oct-2018 | 16.88 | 74729 |
| Pi3 | floor | 38 | 28 | 31-Oct-2018 | 26.01 | 196087 |
| Pi4 | floor | 41 | 28 | 31-Oct-2018 | 23.92 | 75956 |
| Pi5 | floor | 41 | 27 | 31-Oct-2018 | 26.58 | 91192 |
| Pi1 | floor | 45 | 28 | 31-Oct-2018 | 127.80 | 63889 |
| AP1 | seat | 56 | 20 | 29-Oct-2018 | 22.19 | 46070 |
| AP2 | seat | 46 | 28 | 29-Oct-2018 | 2.65 | 64815 |
| AP3 | seat | 50 | 29 | 29-Oct-2018 | 2.77 | 94022 |
| AP4 | seat | 46 | 29 | 29-Oct-2018 | 1.45 | 53354 |
| AP5 | seat | 46 | 29 | 29-Oct-2018 | 17.05 | 26898 |
| AF2 | seat | 50 | 26 | 31-Oct-2018 | 2.63 | 26320 |
| AF3 | seat | 50 | 26 | 31-Oct-2018 | 21.47 | 30490 |
| AF1 | seat | 46 | 26 | 31-Oct-2018 | 18.55 | 68947 |
| AF4 | seat | 52 | 26 | 31-Oct-2018 | 1.62 | 39919 |
| AF5 | seat | 53 | 26 | 31-Oct-2018 | 2.46 | 75340 |
