# Supplementary material for: Passenger-surface microbiome interactions in the subway of Mexico City
Source: PLoS One. 2020 Aug 19;15(8):e0237272. doi: 10.1371/journal.pone.0237272 (PMC7437895; doi:10.1371/journal.pone.0237272)
Supplement: S7 Table — (PDF) [file pone.0237272.s013.pdf]

Table S7. Numbers of raw reads, paired-end reads, and OTUs.

	Datasets	Total (N=89 samples)	Mean by sample	Standard deviation
	Raw reads	10,538,220	118,408	77,582
Sequences	Paired sequences	5,238,317	58,858	38,830
	Paired sequences in OTU table (no singletons)	3,055,072	34,327	24,095
	Paired sequences in OTU table (no singletons, mitochondria and chloroplasts)	3,037,152	34,125	23,906
OTUs	Number of OTUs*	1,205,272	11,860	111,920
	Number of OTUs (no singletons)	74,097	2,668	2,945
	Number of OTUs (no singletons, mitochondria and chloroplasts)	72,817	2,625	2,865
	Number of OTUs from rarefied OTU table	2,9811	801	601

*OTU taxonomic assigment was performed using the GreenGenes database.
