# Supplementary material for: Passenger-surface microbiome interactions in the subway of Mexico City
Source: PLoS One. 2020 Aug 19;15(8):e0237272. doi: 10.1371/journal.pone.0237272 (PMC7437895; doi:10.1371/journal.pone.0237272)
Supplement: S8 Table — (PDF) [file pone.0237272.s014.pdf]

Table S8. Shared genera among samples within the same surface type and among all samples.

Surface types	N	Shared genera
Platform floors	5	148
Escalator hansdrails	5	138
Stair handrails	5	26
Turnstiles	5	66
Poles (regular wagons)	5	49
Poles (women-only wagons)	5	17
Train seats (mix wagons)	5	39
Train seats (women-only wagons)	5	28
Total shared taxa among all samples	40	9*

**Acinetobacter*, *Corynebacterium, Streptococcus, Staphylococcus, Propionibacterium, Kocuria, Pseudomonas,* Micrococcaceae, *Micrococcus*
