# Supplementary material for: Passenger-surface microbiome interactions in the subway of Mexico City
Source: PLoS One. 2020 Aug 19;15(8):e0237272. doi: 10.1371/journal.pone.0237272 (PMC7437895; doi:10.1371/journal.pone.0237272)
Supplement: S9 Table — (PDF) [file pone.0237272.s015.pdf]

Table S9. summary of sequences and ASVs in 89 samples

	Datasets	Total (N=89 samples)	Mean by sample	Standard deviation
Reads	Paired reads in ASV table	1,514,746	17,019	11,604
	Paired sequences in ASV table (no mitochondria and chloroplasts)	1,440,874	16,189	11,132
ASVs*	Number of ASVs	20,783	380	387
	Number of ASVs (no mitochondria and chloroplasts)	20,331	369	376

*Amplicon Sequence Variants
