# Supplementary material for: Passenger-surface microbiome interactions in the subway of Mexico City
Source: PLoS One. 2020 Aug 19;15(8):e0237272. doi: 10.1371/journal.pone.0237272 (PMC7437895; doi:10.1371/journal.pone.0237272)
Supplement: S10 Table — (PDF) [file pone.0237272.s016.pdf]

Table S10. Number of vaginal-associated taxa as a female environmental indicator.

Taxa (ASV)*	Poles		Train seats	
	Regular (N=5)	Women-only (N=5)	Regular (N=5)	Women-only (N=5)
Lactobacillus crispatus	0	0	0	0
Lactobacillus iners AB-1	1	2	1	1
Lactobacillus gasseri	0	0	1	0
Lactobacillus jensenii	0	0	0	0
Atopobium vaginae	0	1	0	0
Sneathia sanguinegens	0	0	0	0
Sneathia amnii	0	0	0	0
Prevotella bivia	1	1	0	1
Prevotella disiens	0	0	0	1
**Sum**	**2**	**4**	**2**	**3**

* Amplicon sequence variants taxonomic assigment was performed using the Silva database.
